# Supplementary material for: A deep-learning system bridging molecule structure and biomedical text with comprehension comparable to human professionals
Source: Nat Commun. 2022 Feb 14;13:862. doi: 10.1038/s41467-022-28494-3 (PMC8844428; doi:10.1038/s41467-022-28494-3)
Supplement: Supplementary file 1 — Reporting Summary [file 41467_2022_28494_MOESM1_ESM.pdf]

nature portfolio

Corresponding author(s): Zhiyuan Liu, Maosong Sun

Last updated by author(s): Jan 11, 2022

Reporting Summary

Nature Portfolio wishes to improve the reproducibility of the work that we publish. This form provides structure for consistency and transparency in reporting. For further information on Nature Portfolio policies, see our Editorial Policies and the Editorial Policy Checklist.

Statistics

For all statistical analyses, confirm that the following items are present in the figure legend, table legend, main text, or Methods section.

n/a	Confirmed	
☐	☒	The exact sample size (*n*) for each experimental group/condition, given as a discrete number and unit of measurement
☐	☒	A statement on whether measurements were taken from distinct samples or whether the same sample was measured repeatedly
☐	☒	The statistical test(s) used AND whether they are one- or two-sided<br>*Only common tests should be described solely by name; describe more complex techniques in the Methods section.*
☐	☒	A description of all covariates tested
☒	☐	A description of any assumptions or corrections, such as tests of normality and adjustment for multiple comparisons
☐	☒	A full description of the statistical parameters including central tendency (e.g. means) or other basic estimates (e.g. regression coefficient) AND variation (e.g. standard deviation) or associated estimates of uncertainty (e.g. confidence intervals)
☐	☒	For null hypothesis testing, the test statistic (e.g. *F*, *t*, *r*) with confidence intervals, effect sizes, degrees of freedom and *P* value noted<br>*Give P values as exact values whenever suitable.*
☒	☐	For Bayesian analysis, information on the choice of priors and Markov chain Monte Carlo settings
☒	☐	For hierarchical and complex designs, identification of the appropriate level for tests and full reporting of outcomes
☒	☐	Estimates of effect sizes (e.g. Cohen's *d*, Pearson's *r*), indicating how they were calculated

Our web collection on statistics for biologists contains articles on many of the points above.

Software and code

Policy information about availability of computer code

Data collection	Data collection is finished with the help of open-source packages:<br>- scispacy==0.4.0
Data analysis	Code is in PyTorch and HuggingFace framework is used for language model, developed on Linux version 4.4.0-31-generic. Data analysis and model training is finished with the help of open-source packages:<br>- torch==1.6.0<br>- transformers>=3.3.1<br>- numpy>=1.19.3<br>- sklearn=0.23.2<br>- tqdm==4.62.1<br>- seqeval==1.2.2<br>- chainer_chemistry==0.7.1<br>- rdkit==2020.09.1<br>-subword_nmt==0.3.7<br>- boto3==1.18.21<br>- requests==2.26.0<br>Code can be accessed from GitHub: https://github.com/thunlp/KV-PLM

For manuscripts utilizing custom algorithms or software that are central to the research but not yet described in published literature, software must be made available to editors and reviewers. We strongly encourage code deposition in a community repository (e.g. GitHub). See the Nature Portfolio guidelines for submitting code & software for further information.

Data

Policy information about availability of data

All manuscripts must include a data availability statement. This statement should provide the following information, where applicable:

- Accession codes, unique identifiers, or web links for publicly available datasets
- A description of any restrictions on data availability
- For clinical datasets or third party data, please ensure that the statement adheres to our policy

The datasets generated and/or analysed during the current study (including ChemProt, BC5CDR, MoleculeNet, USPTO 1k and PCdes) are available from the github link: https://github.com/thunlp/KV-PLM

Field-specific reporting

Please select the one below that is the best fit for your research. If you are not sure, read the appropriate sections before making your selection.

[x] Life sciences [ ] Behavioural & social sciences [ ] Ecological, evolutionary & environmental sciences

For a reference copy of the document with all sections, see nature.com/documents/nr-reporting-summary-flat.pdf

Life sciences study design

All studies must disclose on these points even when the disclosure is negative.

Sample size	Sample sizes are determined according to community consensus, taking full sample size in standard benchmark and 64 samples for each class in few-shot scenario (e.g. 10-shot and 64-shot is tested in "Prompt programming for large language models: Beyond the few-shot paradigm." 2021.).
Data exclusions	No data exclusions.
Replication	The reproducibility can be verified by re-running code provided by us.
Randomization	A variety of random seeds are used and average results are reported. Experiment groups are pre-defined by the previous datasets and randomly split in our own datasets.
Blinding	The investigators are blinded to group allocation during data collection and analysis.

Reporting for specific materials, systems and methods

We require information from authors about some types of materials, experimental systems and methods used in many studies. Here, indicate whether each material, system or method listed is relevant to your study. If you are not sure if a list item applies to your research, read the appropriate section before selecting a response.

Materials & experimental systems

n/a	Involved in the study	
[x]	[ ]	Antibodies
[x]	[ ]	Eukaryotic cell lines
[x]	[ ]	Palaeontology and archaeology
[x]	[ ]	Animals and other organisms
[ ]	[x]	Human research participants
[x]	[ ]	Clinical data
[x]	[ ]	Dual use research of concern

Methods

n/a	Involved in the study	
[x]	[ ]	ChIP-seq
[x]	[ ]	Flow cytometry
[x]	[ ]	MRI-based neuroimaging

Human research participants

Policy information about studies involving human research participants

Population characteristics	The respondents are 6 undergraduates and postgraduates who are studying in top universities who major in chemistry without exam failure record. Other covariate-relevant population characteristics including (e.g. age and gender) are private information irrelevant to their academic level.
Recruitment	We post paid experimental advertisements on the social media platform of the universities to recruit, and check their transcripts to guarantee that they do not fail any exams.
Ethics oversight	This research does not involve ethical issues. Academic Committee of the Department of Computer Science and Technology of Tsinghua University approved the protocol.

Note that full information on the approval of the study protocol must also be provided in the manuscript.
